# Pathological and Molecular Significance of HER2/neu Overexpression in Familial Breast Cancer Among Egyptian Women: A Comprehensive Study on Diagnostic and Prognostic Implications

**DOI:** 10.34172/apb.025.43866

**Published:** 2025-08-02

**Authors:** Amoura M. Abou-El-Naga, Afaf M. El Saied, Maher Monir Akl, Sahar. A. Abd EL-Aziz

**Affiliations:** ^1^Department of Zoology, Faculty of Science, Mansoura University, 35516, Mansoura, Egypt; ^2^Faculty of Medicine, Novosibirsk State University, Russia. B.Sc. in Chemistry, Faculty of Science, Mansoura University, Mansoura, Egypt; ^3^Biochemistry and Molecular Biology Department, Faculty of Medicine, Mansoura University, Mansoura, Egypt

**Keywords:** HER2/neu, ERBB2 amplification, Familial breast cancer, Oncogenic signaling, Immune evasion, Egyptian women

## Abstract

**Purpose:**

Breast cancer remains the most prevalent malignancy among women worldwide, with a strikingly high incidence in Egypt, particularly in familial cases. This study aims to comprehensively elucidate the pathological and molecular significance of ERBB2 (HER2/neu) overexpression in Egyptian familial breast cancer, highlighting its role in tumor aggressiveness, immune evasion, and precision oncology.

**Methods:**

We enrolled 44 Egyptian breast cancer patients along with 35 daughters and 24 sisters (2013–2015, Mansoura University Hospital). Comprehensive analyses included serum biochemical assays, histopathological evaluation, immunohistochemical staining for ERBB2, and molecular detection of ERBB2 amplification using first-round and nested PCR. Associations with clinical, hormonal, and metabolic variables were also explored.

**Results:**

Serum biochemical profiling revealed significantly elevated ALT (6.6±0.55 U/mL), LDH (16.8±1.4 U/mL), and CA15 3 (160±13.33 U/mL), with reduced AST (2.6±0.22 U/mL) compared to controls (*P*≤0.05). Histopathology confirmed invasive ductal carcinoma with dense stromal desmoplasia. Immunohistochemistry demonstrated ERBB2 overexpression in>10% of tumor cells. Nested PCR detected ERBB2 amplification in 72% of patients, and in daughters (17%) and sisters (20%). Notably, higher ERBB2 expression was observed in unmarried patients (100%), pre-menopausal women (73–72%), and those with diabetes or hypertension, suggesting hormonal and metabolic modulation via PI3K/AKT/mTOR and MAPK/ERK pathways. ERBB2 mutations were identified in 14% of patients, 2.1% of daughters, and 1.2% of sisters. Furthermore, ERBB2 may upregulate PD-L1, contributing to immune evasion.

**Conclusion:**

Our findings highlight ERBB2 as a pivotal diagnostic and prognostic biomarker in Egyptian familial breast cancer and support integrating HER2-targeted therapies with immune checkpoint inhibitors and metabolic interventions. This approach could transform outcomes in high-risk familial cohorts. The study emphasizes the importance of genetic screening and precision medicine strategies that consider molecular, hormonal, and metabolic contexts in breast cancer care.

## Introduction

 Breast cancer remains the most prevalent malignancy among women worldwide, with approximately 2.3 million new cases diagnosed annually.^1^ Molecular classification based on mRNA gene expression profiles categorizes breast cancer into distinct subtypes, providing critical insights into novel therapeutic strategies and enabling precise patient stratification for optimized clinical management.^2^ In Egypt, breast cancer accounts for 33% of female cancer cases, with over 22,000 new diagnoses each year.^3^ Age is a well-established determinant of breast cancer etiology, prognosis, and treatment response. The lifetime risk of developing breast cancer is approximately 1 in 8 for women, with over 40% of cases occurring in those aged 65 and older, who contribute to nearly 60% of breast cancer-related mortality.^4^ For women under 49, the risk is 1 in 53, rising to 1 in 43 for those aged 50–59, 1 in 23 for those aged 60–69, and peaking at 1 in 15 for women over 70.^4^ The human epidermal growth factor receptor 2 (HER2/neu), encoded by the ERBB2 gene on chromosome 17q, is a transmembrane receptor tyrosine kinase that regulates cell growth, division, and repair under normal physiological conditions.^5^

 In 20–30% of breast cancer cases, amplification or overexpression of ERBB2 leads to excessive HER2 protein production, driving aggressive tumor behavior, accelerated proliferation, and poor prognostic outcomes.^5^ HER2/neu overexpression activates key oncogenic signaling pathways, including the PI3K/AKT/mTOR and MAPK/ERK cascades, which promote uncontrolled cell proliferation, survival, and resistance to apoptosis.^6^ Additionally, HER2/neu modulates the tumor microenvironment by enhancing immunosuppressive mechanisms, such as upregulation of programmed death-ligand 1 (PD-L1) and recruitment of regulatory T cells, thereby attenuating anti-tumor immune responses.^7^ This dual role in oncogenic signaling and immune evasion underscores HER2/neu’s significance in tumor progression.^8^ In familial breast cancer, where first-degree relatives face an elevated risk, HER2/neu amplification is frequently associated with more aggressive disease phenotypes.^9^

 This study investigates the pathological and molecular significance of HER2/neu overexpression in familial breast cancer among Egyptian women. Through comprehensive histopathological, immunohistochemical, and molecular analyses, we aim to elucidate HER2/neu’s role as a therapeutic target and prognostic indicator. By exploring its contributions to oncogenic signaling and immune modulation, our findings seek to advance the understanding of HER2/neu in familial breast cancer and inform precision-based clinical interventions.

## Materials and Methods

###  Study population and sample collection

 This study was conducted in compliance with the ethical guidelines of the Faculty of Medicine, Mansoura University, Egypt, and approved by its Institutional Review Board. Written informed consent was obtained from all participants prior to enrollment. A cohort of 44 female breast cancer patients, aged 40–60 years, was recruited from the Department of Surgery, Mansoura University Hospital, between 2013 and 2015. Additionally, 35 daughters and 24 sisters of these patients were included for familial analysis. Venous blood samples (5 mL) were collected from each participant and divided into two aliquots: 3 mL for serum isolation, subsequently analyzed by enzyme-linked immunosorbent assay (ELISA), and 2 mL stored with anticoagulant at -20 °C for DNA extraction and ERBB2 (HER2/neu) polymorphism analysis.

###  Chemicals and reagents

 All chemicals and reagents were of analytical grade and procured from Sigma-Aldrich (St. Louis, MO, USA). Reagents for biochemical and molecular analyses included 0.2 M Tris-HCl buffer (pH 7.3), 6.6 mM NADH in 0.2 M Tris-HCl buffer, and 30 mM sodium pyruvate in 0.2 M Tris-HCl buffer, prepared according to standard protocols.

###  Biochemical analysis

 Serum levels of aspartate aminotransferase (AST), alanine aminotransferase (ALT), and lactate dehydrogenase (LDH) were quantified using RAM diagnostic kits (RAM Diagnostics, Egypt). AST activity was measured by the enzymatic reaction of α-oxoglutarate with L-aspartate to produce L-glutamate and oxaloacetate, with oxaloacetate hydrazone formed using 2,4-dinitrophenylhydrazine and quantified spectrophotometrically against a standard curve. ALT activity was assessed using the Reitman and Frankel method, measuring L-glutamate and pyruvate production colorimetrically. LDH activity was determined by monitoring the decrease in absorbance at 340 nm due to NADH oxidation, with one unit defined as the oxidation of 1 μmol of NADH per minute at 25 °C and pH 7.3.

###  Serum HER2/neu quantification

 Serum HER2/neu extracellular domain levels were quantified using the c-erbB-2/c-neu Rapid Format ELISA kit (Calbiochem, USA). Microtiter wells coated with anti-p185 murine monoclonal antibodies were used, with recombinant p185 HER2/neu protein as the standard. Biotinylated murine anti-human c-erbB-2/c-neu served as the detection antibody, and tetramethylbenzidine (TMB) was used as the chromogenic substrate. Absorbance was measured at 450/595 nm using a microplate reader.

###  Molecular analysis

 Genomic DNA was extracted from whole blood using the GeneJET DNA Purification Kit (Thermo Fisher Scientific, Canada) following lysis with proteinase K. DNA integrity was verified by electrophoresis on a 1% agarose gel stained with ethidium bromide (0.5 μg/mL) and visualized under UV light. Amplification of the ERBB2 gene was performed using DreamTaq Green PCR Master Mix (Thermo Fisher Scientific) with primers specific to ERBB2. PCR products were analyzed on 3% agarose gels stained with ethidium bromide and visualized using a UV transilluminator, with a 100–1000 bp DNA ladder (SibEnzyme, Russia) as a reference.

###  Agarose gel electrophoresis

 A 3% agarose gel was prepared in 1X Tris-EDTA (TE) buffer, cooled, and stained with ethidium bromide (0.5 μg/mL). DNA samples, mixed with loading buffer, were electrophoresed at 100 V for 30 minutes. DNA bands were visualized under UV light, with a 100–1000 bp DNA ladder (SibEnzyme, Russia) for size reference. A stock solution of ethidium bromide (1 g in 200 mL double-distilled water) was prepared and stored at 4°C in a light-protected container.

###  Polymerase chain reaction (PCR) amplification

 To amplify the HER2/neu gene, genomic DNA was first purified and subjected to PCR using specific primer pairs.

 The primers used for the first PCR were as follows:

- Forward: ATA TCC AGG AGG TGC AGG G
- Reverse: CTT CGA AGC TGC AGC TCC C

Each reaction was prepared in a final volume of 25 µL, comprising 12 µL of 2 × Green master mix, 4 µL of forward primer, 4 µL of reverse primer, and 5µL of DNA template. The reaction mixture was gently mixed by pipetting up and down and loaded into 0.2 mL Eppendorf tubes. Tubes were sealed tightly before placement in the thermal cycler.

 The thermal cycling protocol included an initial denaturation step at 96 °C for 10 minutes, followed by 35 cycles consisting of denaturation at 96 °C for 1 minute, annealing at 53 °C for 1 minute, and extension at 72 °C for 1 minute. PCR products of 205 bp were visualized by agarose gel electrophoresis. For sequence verification, PCR products were purified and concentrated using Amicon Ultrafree-DA membranes (Millipore Corporation, Bedford, MA) according to the manufacturer’s instructions. The amplicons were then sequenced by dye-terminator chemistry using the ABI 377 Sequencer (Applied Biosystems, Foster City, CA). External standards of HER2/neu and β-globin templates were combined in equal concentrations to ensure quality control.

 Following the first amplification, a nested PCR was performed to increase detection sensitivity. In this reaction, 4µL of product from the first PCR served as the template.

 The nested PCR primer sequences were:

- Forward: CTC ACA ACC AAG TGA GGC AG
- Reverse: CAG GGG TGG TAT TCT TCA

Each nested PCR reaction was prepared in a final volume of 25 µL, containing 10µL of PCR product from the first amplification, 3 µL of forward primer, 3 µL of reverse primer, and 9 µL of 2 × master mix. The mixture was gently mixed by pipetting, loaded into 0.2 ml Eppendorf tubes, and securely closed.

 The thermal cycler was programmed with an initial denaturation at 96 °C for 10 minutes, followed by 35 cycles comprising denaturation at 96 °C for 1 minute, annealing at 58 °C for 1 minute, and extension at 72 °C for 1 minute. The nested PCR products, expected at 126 bp, were visualized by agarose gel electrophoresis. Overall, the annealing temperature was adjusted to 53 °C for the first PCR and 58 °C for the nested PCR to optimize specificity and amplification efficiency.

 This two-step amplification strategy enabled sensitive and specific detection of HER2/neu gene expression in breast cancer patients and their first-degree relatives.

###  Histopathological and immunohistochemical analysis of breast tissue for HER2/neu expression

 Following surgical resection of breast tissue, biopsies were sectioned into small fragments and immediately fixed in 10% neutral buffered formalin (pH 7.4) for 24 hours. The tissues were dehydrated through an ascending series of ethanol concentrations, cleared in xylene, and embedded in molten paraffin at 58–60 °C. Sections of 5 μm thickness were prepared using a microtome, mounted on glass slides, and stained with hematoxylin and eosin (H&E) for histopathological examination. The stained sections were examined under a bright-field Olympus microscope. For immunohistochemical analysis, paraffin-embedded sections were deparaffinized in xylene and rehydrated through a descending series of ethanol concentrations. Endogenous peroxidase activity was quenched following the manufacturer’s protocol (Dako, Germany), and antigen retrieval was performed using citrate buffer (pH 6.0). Sections were incubated with a diluted monoclonal anti-HER2/neu antibody (CB11, Novocastra, Newcastle, UK). Positive reactions were developed using the EnVision + System (Dako) with diaminobenzidine (DAB) as the chromogenic substrate. The specimens were counterstained with Mayer’s hematoxylin, dehydrated, cleared in xylene, mounted in DPX, and examined under a bright-field Olympus microscope equipped with a Canon digital camera. HER2/neu oncoprotein overexpression was defined by strong, complete membrane staining. For negative controls, the primary antibody was replaced with phosphate-buffered saline (PBS), while all other steps remained identical. Microscopic evaluation was performed to assess staining intensity and distribution.

###  Statistical Analysis

 Data were analyzed using SPSS version 20.0 (IBM Corp., Armonk, NY, USA). The normality of continuous variables was assessed using the Kolmogorov-Smirnov test, confirming a normal distribution. Continuous variables were summarized using means and standard deviations to describe central tendency and dispersion. Differences in continuous variables between two groups were evaluated using an independent samples t-test.

 For categorical variables, data were expressed as numbers and percentages, with differences between groups analyzed using the Chi-square test. Fisher’s exact test was applied for continuity correction when expected cell counts were low, and odds ratios were calculated to estimate risk. A p-value of less than 0.05 was considered statistically significant for all analyses.

## Results

**Figure 1:**
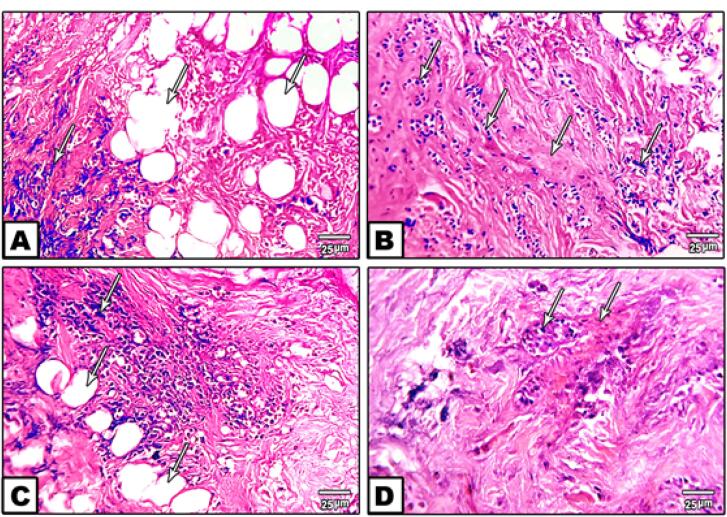
Photomicrographs of breast cancer tissue stained with hematoxylin and eosin (H&E). Panels A and B illustrate invasive cancer cells within glandular ducts accompanied by stromal desmoplasia. Panels C and D depict round to oval nuclei with minimal mitotic activity and irregular invasive glands eliciting a dense desmoplastic reaction in the surrounding stroma, indicative of aggressive tumor morphology

 Breast cancer patients demonstrated significantly elevated serum levels of ALT (6.6 ± 0.55 U/mL vs. 4.2 ± 0.45 U/mL in controls), LDH (16.8 ± 1.4 U/mL vs. 9.4 ± 0.78 U/mL), and cancer antigen 15-3 (CA15-3; 160 ± 13.33 U/mL vs. 33.5 ± 2.79 U/mL), alongside a marked reduction in AST (2.6 ± 0.22 U/mL vs. 5.8 ± 0.54 U/mL), with all differences statistically significant (*P* ≤ 0.05). Serum HER2/neu extracellular domain levels were also significantly elevated (0.12 ng/mL vs. 0.06 ng/mL in controls), underscoring its diagnostic potential (Table 1). Histopathological examination of breast tissue biopsies revealed intra-epithelial spaces of varying sizes, invasive cancer cells within glandular ducts, round to oval nuclei with minimal mitotic activity, and dense stromal desmoplasia, indicative of aggressive tumor behavior (Figure 1).

**Table 1:** Biochemical and tumor markers in breast cancer patients vs. controls

**Marker**	**Controls (n=10)**	**Breast cancer patients (n=10)**	***P*** ** value**
ALT (U/mL)	4.2 ± 0.45	6.6 ± 0.55 *	≤ 0.05
AST (U/mL)	5.8 ± 0.54	2.6 ± 0.22 *	≤ 0.05
LDH (U/mL)	9.4 ± 0.78	16.8 ± 1.4 *	≤ 0.05
Cancer antigen 15-3 (CA15-3) (U/mL)	33.5 ± 2.79	160 ± 13.33 *	≤ 0.05
HER2/neu protein (ng/mL)	0.062	0.12 *	≤ 0.05

* Statistically significant compared with controls (*P* ≤ 0.05). Values are presented as mean ± standard error.

**Figure 2:**
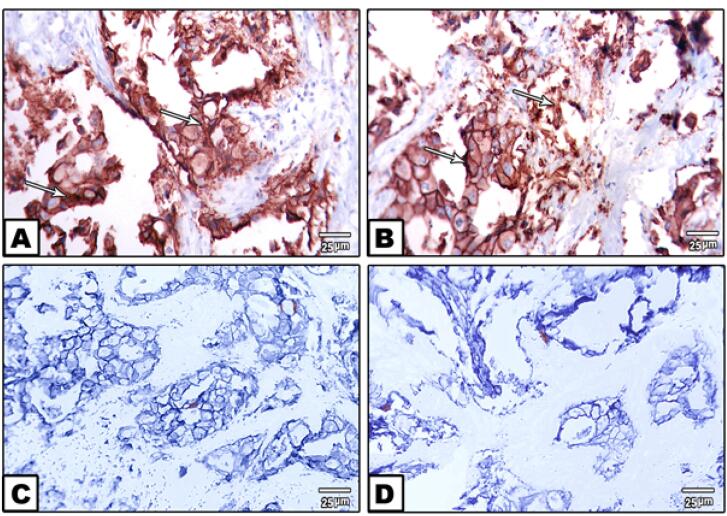
Immunohistochemical staining of paraffin-embedded breast cancer tissue sections using anti-HER2/neu antibody. Panels A and B demonstrate strong, dark-brown membrane staining indicative of HER2/neu oncoprotein overexpression in invasive ductal carcinoma cells. Panels C and D show regions of breast cancer tissue lacking HER2/neu expression, highlighting heterogeneous protein distribution within the tumor

**Figure 3:**
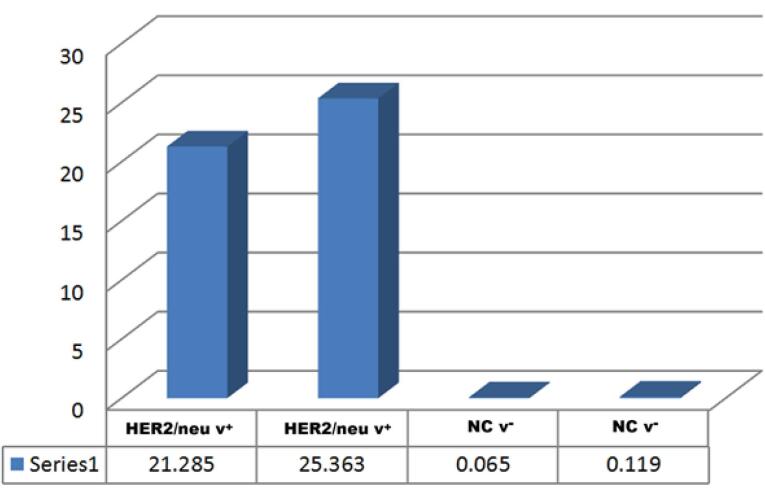
Chart illustrating quantitative image analysis of immunohistochemical staining for ERBB2 (HER2/neu) expression in breast cancer tissue. The data reveal that estrogen receptor (ER) scores of 4 + and 7 + were observed in approximately 30% (weak positive) and 80% (moderate positive) of tumor cells, respectively, by nuclear staining. Progesterone receptor (PR) scores of 7 + were detected in 80% of tumor cells, indicating significant hormonal receptor expression

 Immunohistochemical analysis showed strong, complete membrane staining for HER2/neu oncoprotein in invasive ductal carcinoma cells, with over 10% of tumor cells assigned a HER2/neu score of + 3, reflecting ERBB2 gene amplification. In contrast, other regions exhibited minimal to no HER2/neu expression (Figure 2). Image analysis further confirmed elevated ERBB2 expression, with estrogen receptor (ER) scores of 4 + and 7 + observed in approximately 30% (weak positive) and 80% (moderate positive) of tumor cells, respectively, and progesterone receptor (PR) scores of 7 + in 80% of tumor cells by nuclear staining (Figure 3). Molecular analysis using first-round and nested PCR revealed ERBB2 amplification in 57% and 72% of patients, respectively. Among daughters (n = 36), ERBB2 amplification was detected in 80% (first PCR) and 17% (nested PCR), while among sisters (n = 24), the frequencies were 79% and 20%, respectively (Table 2).

**Table 2:** HER2/neu gene expression by PCR and nested PCR in patients and family members

**Group**	**Sample size**	**1st PCR positive (%)**	**Nested PCR positive (%)**
Breast cancer patients	44	25 (57%)	32 (72%)
Daughters	36	29 (80%)	6 (17%)
Sisters	24	19 (79%)	5 (20%)
Married women	40	21 (52%)	29 (72%)
Non-married women	4	4 (100%)	4 (100%)
Post-menstrual women	19	9 (42%)	15 (62%)
Pre-menstrual women	25	15 (73%)	18 (72%)
ER-negative patients	23	13 (56%)	17 (73%)
ER-positive patients	18	10 (55%)	14 (77%)
PR-negative patients	22	13 (59%)	16 (72%)
PR-positive patients	19	10 (52%)	15 (72%)
Non-diabetic patients	31	16 (51%)	24 (77%)
Diabetic patients	6	4 (66%)	4 (66%)
Non-hypertensive patients	30	17 (56%)	21 (70%)
Hypertensive patients	8	3 (37%)	8 (100%)

Table 2 illustrates the distribution of HER2/neu gene expression detected by first PCR and nested PCR among breast cancer patients and their first-degree female relatives (daughters and sisters). The table further explores the association of HER2/neu positivity with clinicopathological and demographic variables, including marital status, menopausal status, hormone receptor status (ER and PR), diabetes, and hypertension. The nested PCR consistently demonstrated higher detection rates compared to the first PCR, reflecting its increased sensitivity in identifying HER2/neu overexpression across different patient subgroups.

 Notably, ERBB2 mutations were identified in 14% of patients (32/44), 2.1% of daughters (6/35), and 1.2% of sisters (5/24) (Table 3).

**Table 3:** Frequency of HER2/neu Gene Mutations Identified by Nested PCR

**Group**	**Total number**	**Number with mutation**	**Frequency (%)**
Breast cancer patients	44	32	14%
Daughters	35	6	2.1%
Sisters	24	5	1.2%

Table 3 summarizes the frequency of HER2/neu gene mutations identified by nested PCR among breast cancer patients, daughters, and sisters. The data reveal a markedly higher mutation rate among patients (14%) compared to daughters (2.1%) and sisters (1.2%), highlighting the familial distribution and potential hereditary predisposition associated with HER2/neu alterations.

**Figure 4:**
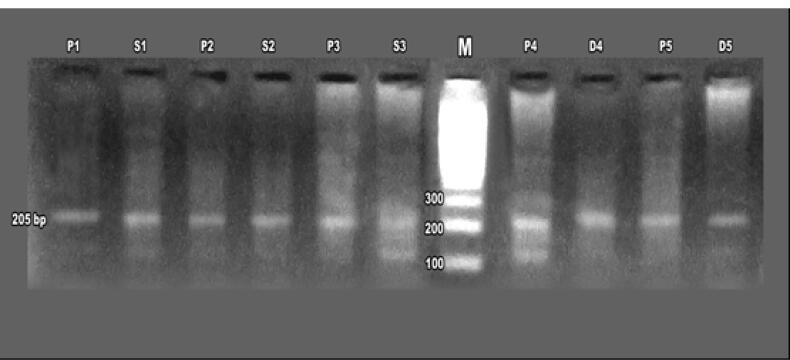
Agarose gel electrophoresis of first-round PCR products amplifying the ERBB2 (HER2/neu) gene from breast cancer patients (P1–P5), daughters (D4, D5), and sisters (S1–S3). The gel includes a 100 bp DNA ladder (Thermo Fisher Scientific) for size reference, demonstrating the presence of ERBB2 amplicons in patient and familial samples

**Figure 5:**
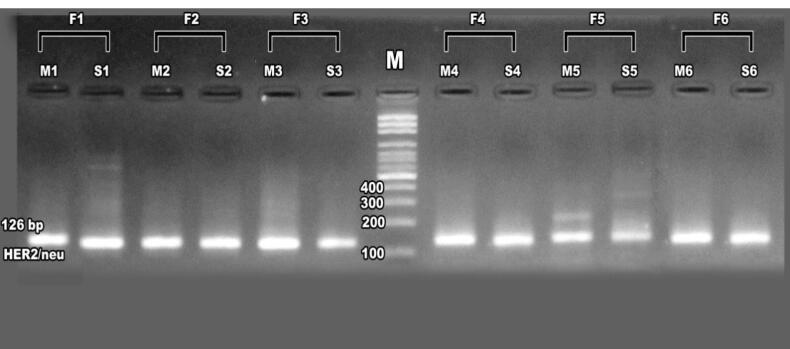
Agarose gel electrophoresis of nested PCR products amplifying the ERBB2 (HER2/neu) gene at 126 bp from familial breast cancer cases (F1–F6), including mothers (M1–M6) and sisters (S1–S6). A 100 bp DNA ladder (Thermo Fisher Scientific) serves as a size reference, confirming ERBB2 amplification in familial samples

**Figure 6:**
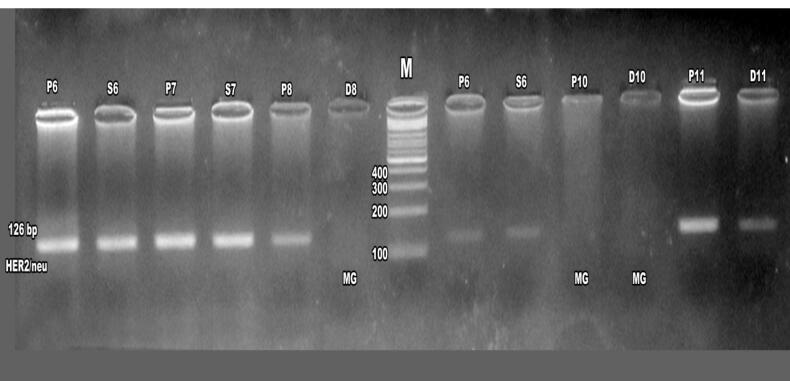
Agarose gel electrophoresis of nested PCR products amplifying the ERBB2 (HER2/neu) gene at 126 bp from breast cancer patients (P6–P11), daughters (D8, D10), and sisters (S6–S8). A 100 bp DNA ladder (Thermo Fisher Scientific) is included for size reference. The absence of ERBB2 amplicons in some samples suggests potential mutations affecting gene expression

**Figure 7:**
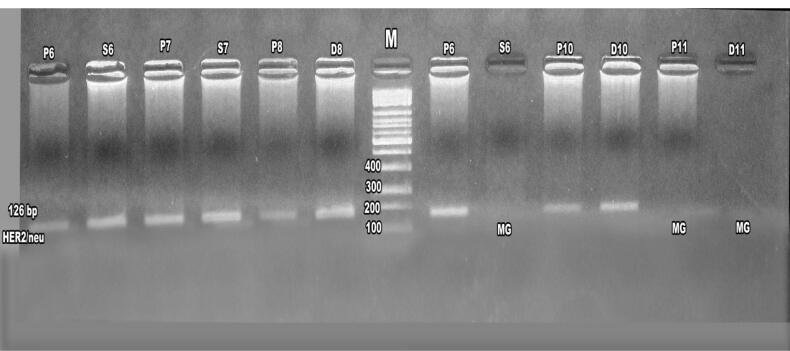
Agarose gel electrophoresis of nested PCR products amplifying the ERBB2 (HER2/neu) gene at 126 bp from breast cancer patients (P6–P11), daughters (D8, D10, D11), and sisters (S6–S8). A 100 bp DNA ladder (Thermo Fisher Scientific) is included for size reference. Notably, absent ERBB2 amplicons in samples from S6, P11, and D11 indicate potential mutations disrupting ERBB2 expression

 Agarose gel electrophoresis of first-round PCR products confirmed ERBB2 amplification in patients and their daughters and sisters, (Figure 4) with nested PCR showing amplified products at 126 bp in familial cases, including mothers, daughters, and sisters, though some cases exhibited absent ERBB2 expression, suggesting potential mutations (Figures 5-7). ERBB2 overexpression was significantly higher in unmarried patients (100% in both PCR assays) compared to married patients (52% in first PCR, 72% in nested PCR), and in pre-menopausal patients (73% in first PCR, 72% in nested PCR) compared to post-menopausal patients (42% in first PCR, 62% in nested PCR). Patients with diabetes (66% in both PCR assays) and hypertension (37% in first PCR, 100% in nested PCR) also showed elevated ERBB2 expression compared to those without these conditions. These findings suggest that ERBB2 overexpression activates oncogenic signaling pathways, such as PI3K/AKT/mTOR and MAPK/ERK, promoting tumor proliferation and survival, particularly in patients with metabolic comorbidities. Additionally, ERBB2 may modulate the tumor microenvironment by upregulating programmed death-ligand 1 (PD-L1), potentially contributing to immune evasion, which could explain its association with aggressive disease phenotypes in familial breast cancer and specific clinical factors such as hormonal status and chronic conditions.

## Discussion

 This study provides comprehensive insights into the biochemical, histopathological, immunohistochemical, and molecular characteristics of familial breast cancer among Egyptian women, highlighting the pivotal role of ERBB2 (HER2/neu) overexpression in disease pathogenesis and its potential as a diagnostic and prognostic biomarker. The significant elevation of serum ALT (6.6 ± 0.55 U/mL), LDH (16.8 ± 1.4 U/mL), and cancer antigen 15-3 (CA15-3; 160 ± 13.33 U/mL), alongside reduced AST (2.6 ± 0.22 U/mL) in breast cancer patients compared to controls (Table 1), suggests a metabolic shift consistent with the Warburg effect, where tumor cells favor glycolysis to support rapid proliferation.^10,11^ Elevated LDH levels likely reflect increased glycolytic activity and mitochondrial stress, common in aggressive tumors,^12^ while reduced AST may indicate altered hepatic function or tissue-specific metabolic changes.^13^ The marked increase in serum HER2/neu extracellular domain (0.12 ng/mL vs. 0.06 ng/mL in controls) underscores its utility as a non-invasive biomarker for monitoring disease progression and therapeutic response.^14^

 Histopathologically, the presence of intra-epithelial spaces, invasive ductal carcinoma cells, and dense stromal desmoplasia (Figure 1) confirms the aggressive nature of the tumors in our cohort.^15^ Immunohistochemical analysis revealed strong, complete membrane staining for HER2/neu in over 10% of tumor cells, with estrogen receptor (ER) and progesterone receptor (PR) scores indicating significant hormonal influence (80% moderate positive for ER and PR) (Figures 2 and 3). These findings align with the established role of ERBB2 amplification in driving tumor aggressiveness through activation of oncogenic signaling pathways, including PI3K/AKT/mTOR and MAPK/ERK.^16,17^ These pathways promote cell proliferation, survival, and resistance to apoptosis, contributing to the poor prognosis associated with HER2-positive breast cancers.^18,19^ Furthermore, ERBB2 overexpression may modulate the tumor microenvironment by upregulating programmed death-ligand 1 (PD-L1), facilitating immune evasion and enhancing tumor progression, particularly in familial cases with genetic predispositions.^20^

 Molecularly, the detection of ERBB2 amplification in 57% (first PCR) and 72% (nested PCR) of patients, with frequencies of 80% and 17% in daughters and 79% and 20% in sisters, respectively (Table 2), underscores the hereditary component of ERBB2-driven breast cancer.^21^

 The identification of ERBB2 mutations in 14% of patients, 2.1% of daughters, and 1.2% of sisters (Table 3) suggests a genetic basis for increased susceptibility in first-degree relatives, particularly in populations with high familial breast cancer prevalence, such as in Egypt. Notably, the absence of ERBB2 expression in some familial cases (Figures 6 and 7) may indicate mutations disrupting gene function, warranting further investigation into specific ERBB2 variants.^22^

 A striking observation was the differential ERBB2 expression based on clinical and social factors. Unmarried patients exhibited 100% ERBB2 positivity in both PCR assays, compared to 52% (first PCR) and 72% (nested PCR) in married patients (Table 2). This discrepancy may be attributed to hormonal fluctuations, as pre-menopausal patients showed higher ERBB2 expression (73% in first PCR, 72% in nested PCR) than post-menopausal patients (42% and 62%, respectively). Estrogen and progesterone, known to regulate ERBB2 transcription, likely contribute to these differences, with ER-negative tumors exhibiting more aggressive phenotypes.^23,24^ Additionally, patients with diabetes (66% positivity) and hypertension (37% in first PCR, 100% in nested PCR) displayed elevated ERBB2 expression compared to those without these comorbidities (Table 2). These findings suggest that chronic inflammation and oxidative stress associated with metabolic disorders may amplify ERBB2-mediated oncogenic signaling, potentially through enhanced activation of PI3K/AKT/mTOR and MAPK/ERK pathways.^25,26^ The interplay between metabolic stress and ERBB2 overexpression may also promote an immunosuppressive tumor microenvironment, further exacerbating tumor progression.^11,27,28^

 The high prevalence of ERBB2 amplification in familial breast cancer underscores the importance of genetic screening for first-degree relatives, particularly in high-risk populations. The association of ERBB2 overexpression with hormonal and metabolic factors highlights the need for personalized therapeutic strategies, such as combining HER2-targeted therapies (e.g., trastuzumab) with anti-inflammatory or metabolic interventions.^29^ Moreover, the potential role of ERBB2 in upregulating PD-L1 suggests that combining HER2-targeted therapies with immune checkpoint inhibitors could enhance treatment efficacy in HER2-positive familial breast cancer.^20^ These insights pave the way for precision medicine approaches tailored to the molecular and clinical profiles of Egyptian patients, addressing both genetic and environmental risk factors.

## Conclusion

 In conclusion, our study reinforces the importance of HER2/neu as a critical biomarker for both the diagnosis and prognosis of breast cancer. The overexpression of HER2/neu in both sporadic and familial cases highlights its utility in identifying high-risk individuals, particularly among family members of breast cancer patients.

 Moreover, the association between HER2/neu and hormonal and metabolic factors underscores the need for personalized treatment strategies that consider not only the tumor biology but also the patient’s overall health status. Future studies should focus on long-term follow-up to assess the prognostic implications of HER2/neu overexpression, particularly in relation to early relapse and response to targeted therapies. Genetic counseling and regular screening for family members of breast cancer patients are also recommended to improve early detection and prevention strategies.

## Competing Interests

 The authors declare that they have no known competing financial interests or personal relationships that could have appeared to influence the work reported in this paper.

## Ethical Approval

 This study was conducted in full accordance with the ethical principles of the Declaration of Helsinki and complies with the CARE guidelines for case reports. Written informed consent was obtained from the patients, including explicit permission to publish relevant clinical data, laboratory results, and all photographic or imaging materials presented in this manuscript.

## Informed Consent

 Prior to the inclusion of these cases in the study, comprehensive information regarding the research objectives and procedures was provided to the patients. Written informed consent was subsequently obtained, including consent for clinical follow-up and for publication of related data, figures, and laboratory investigations.
